# A Discrete Ligand-Free T3 Supertetrahedral Cluster of Gallium Sulfide

**DOI:** 10.3390/molecules26175415

**Published:** 2021-09-06

**Authors:** Sarah Makin, Paz Vaqueiro

**Affiliations:** Department of Chemistry, University of Reading, Whiteknights, Reading RG6 6DX, UK; sarahmakin90@hotmail.co.uk

**Keywords:** supertetrahedral cluster, gallium sulfide, crystal structure

## Abstract

Large discrete supertetrahedral clusters of metal chalcogenides are rare due to the difficulty of crystallizing solids in which the negative charge of the cluster is balanced by the positive charges of the countercations. Here, we describe a discrete ligand-free T3 supertetrahedral cluster, [Ga_10_S_16_(SH)_4_]^6−^, which was successfully synthesized in the presence of the superbase 1,8-diazabicyclo[5.4.0]undec-7-ene (DBU) using the neutral surfactant polyethyleneglycol (PEG)-400 as the reaction solvent. Protonated DBUH^+^ cations are incorporated into the crystal structure of the product, which can be formulated as [C_9_H_17_N_2_]_6_[Ga_10_S_16_(SH)_4_]. This compound, which represents the first example of a discrete ligand-free T3 cluster of gallium sulfide, was fully characterized by single-crystal and powder X-ray diffraction, elemental analysis, infrared spectroscopy, thermogravimetric analysis, and ultraviolet-visible diffuse reflectance. The results presented here indicate that the use of surfactants as solvents offers potential for the preparation of new compounds containing supertetrahedral clusters.

## 1. Introduction

Tetrahedral chalcogenide nanoclusters are attractive as building blocks for multifunctional open-framework materials, in which porosity is integrated with semiconducting behavior [1,2,3]. Moreover, as the structures of these nanoclusters are closely related to those of II−VI or I−III−VI semiconductor nanocrystals, chalcogenide nanoclusters can be considered as ultrasmall quantum dots [2,4]. The uniform sizes and well-defined structures of tetrahedral chalcogenide nanoclusters can be advantageous for the investigation of the site- or size-dependent properties of quantum dots [5].

There are four main classes of tetrahedral chalcogenide clusters: supertetrahedral (T*n*), penta-supertetrahedral (P*n*), capped supertetrahedral (C*n*), and super-supertetrahedral (T*p*,*q*) clusters (where *n*, *p*, and *q* are integers which indicate the cluster size) [6,7]. These clusters are formed by chalcogenides of main group elements (Ga, In, Ge, Sn), with transition-metal elements (Fe, Co, Zn, Cd) often included in the larger clusters in order to reduce the overall cluster charge.

In the case of gallium sulfides, the compounds described to date are primarily based on T*n* clusters, which are tetrahedrally shaped fragments of the zinc-blende structure, with *n* indicating the number of tetrahedra along the cluster edge [6,7]. Gallium-sulfide supertetrahedral clusters can be ligand-free or have ligands covalently attached to the four vertexes. For ligand-free clusters, a T1 cluster would correspond to a single tetrahedron [GaS_4_]^5−^, T2 clusters are adamantane units, [Ga_4_S_10_]^8−^, and T3 clusters have the composition [Ga_10_S_20_]^10−^. Discrete ligand-free T2 supertetrahedral clusters, [Ga_4_S_10_]^8−^, were first described by Krebs et al. [8], while a ligand-containing T2 analogue, (NH_3_)_4_Ga_4_S_6_, has been reported very recently [9]. Larger discrete supertetrahedral clusters, such as T3 [Ga_10_S_16_(NC_7_H_9_)_4_]^2−^ (where NC_7_H_9_ = 3,5-dimethylpyridine) [10] and T5 [Cu_5_Ga_30_S_52_(SH)_2_(Bim)_2_]^11−^ (where Bim = 1-butyl-2-methyl-imidazole) [11], are rare and usually have some or all of the sulfur anions at the vertexes of the cluster replaced by organic ligands. Alternatively, as exemplified by the T4 [Mn_4_Ga_14_Sn_2_S_35_]^12−^ and T5 [Cu_5_Ga_30_S_52_(SH)_4_]^13−^ clusters [11,12], large discrete clusters contain divalent or monovalent cations. Either of these two approaches reduces the overall negative charge of the supertetrahedral cluster, hence facilitating its crystallization. Although discrete ligand-free T3 clusters, [Ga_10_S_20_]^10−^, have not been reported to date, these T3 clusters are frequently found as building blocks of gallium sulfide open-framework structures, as exemplified by [amineH^+^]_6_[Ga_10_S_18_] (where amine = diethylamine, 1-(2-aminoethyl)piperazine, and ethanolamine), in which vertex-linked T3 clusters form an interpenetrating double-diamond lattice [9,13,14]. Similarly, organically functionalized T3 clusters are frequently found in hybrid extended structures [15,16,17].

Discrete supertetrahedral clusters are attractive as ultrasmall quantum dots [5] and as potential precursors for the synthesis of open-framework chalcogenides [18]. However, discrete ligand-free clusters are very rare due to the difficulty in balancing the large negative charge of the cluster with the positive charges of the countercations. Here, we present the synthesis and characterization of the first ligand-free discrete T3 cluster, [Ga_10_S_16_(SH)_4_]^6−^, which was facilitated through the incorporation of a neutral surfactant, PEG-400, into the reaction mixture.

## 2. Results

### 2.1. Crystal Structure of [C_9_H_17_N_2_]_6_[Ga_10_S_16_(SH)_4_]

**Figure 1:**
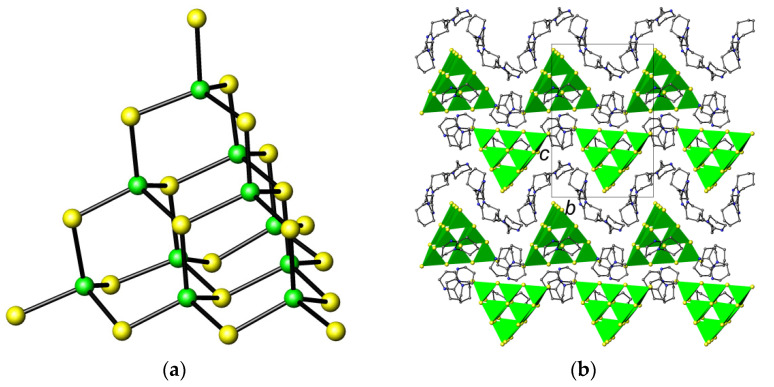
(**a**) The T3 supertetrahedral cluster found in [C_9_H_17_N_2_]_6_[Ga_10_S_16_(SH)_4_]. Color key: green spheres, Ga; yellow spheres, S. (**b**) View of the crystal structure along the *a*-axis. Unit cell is shown. Color key: GaS_4_, green tetrahedra; yellow spheres, S; blue spheres, N; grey spheres, C. H atoms have been omitted for clarity.

Selected crystallographic information is summarised in Table 1. The asymmetric unit of the title compound contains a discrete T3 gallium sulfide supertetrahedron (Figure 1a) and six DBU moieties. Unlike previously reported T3 gallium sulfide clusters [10], the cluster found here is ligand-free.

As each of the six DBU moieties found per formula unit can only be monoprotonated, four further protons are needed in order to balance the charge and hence the four sulfur atoms at the vertexes of the cluster must be SH*^−^* units. Elemental analysis (experimental: C = 28.62%; H = 4.68%; N = 7.30%; calculated: C = 28.67%; H = 4.72%; N = 7.43%) was in excellent agreement with the proposed formula, [C_9_H_17_N_2_]_6_[Ga_10_S_16_(SH)_4_].

The Ga–S distances in the T3 cluster lie in the range of 2.216(2)–2.336(2) Å, with the longest distances corresponding to the µ_3_-S atoms at the center of each supertetrahedral face, whereas those in -Ga-S-H groups are in the range of 2.276(3)–2.294(3) Å. These values are comparable to those found in previously reported gallium sulfides containing T3 supertetrahedral clusters [13], as well as in those containing terminal -Ga-S-H groups [11]. In the crystal structure of the title compound, the T3 supertetrahedra are packed in layers parallel to the (110) planes (Figure 1b). The N-S distances between the DBUH^+^ cations and the T3 clusters, which range between 3.216(7) and 3.458(11) Å, are consistent with hydrogen-bonding interactions. In addition, there are also a number of short contacts of the type C-H…S, which result in the formation of a three-dimensional supramolecular network.

### 2.2. Characterisation

**Figure 2:**
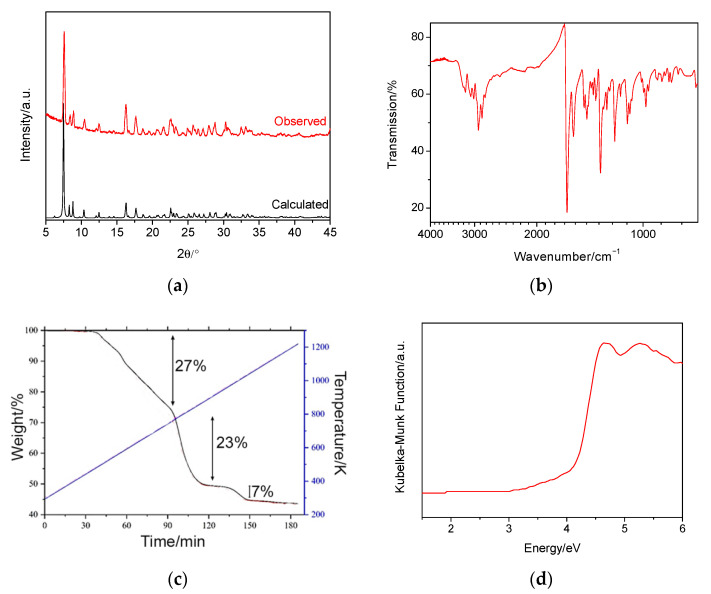
(**a**) Powder X-ray diffraction data collected for ground crystals of [C_9_H_17_N_2_]_6_[Ga_10_S_16_(SH)_4_]. (**b**) FT-IR data. (**c**) Thermogravimetric analysis data, collected under air. The temperature is given by the blue line. (**d**) UV–Vis diffuse reflectance data.

The powder X-ray diffraction pattern collected on ground crystals of the title compound is in good agreement with the simulated powder pattern generated from the crystal structure determined by single-crystal diffraction (Figure 2a). The FT-IR data (Figure 2b), which are comparable to those reported for the [DBUH][SH] salt [19], are consistent with the presence of protonated DBUH^+^ cations. For instance, there are C-H stretches around 2900 cm*^−^*^1^, as well as C-H bends around 1400 cm*^−^*^1^. Vibrational bands found between 3200 and 3000 cm*^−^*^1^ can be assigned to N-H stretches, while those found at 1630 and 1570 cm*^−^*^1^ are in the C=N ring stretching vibration range. A very weak feature at 2550 cm*^−^*^1^ can be attributed to the strech of the S-H groups in the supertetrahedral cluster.

The thermogravimetric analysis (Figure 2c), carried out by heating this material in air, can be interpreted as follows. The first weight change corresponds to the loss of four protonated DBUH^+^ cations, [C_9_H_17_N_2_]^+^. The second weight-loss step corresponds to the removal of the remaining two DBUH^+^ cations, together with five S atoms per formula unit, to form Ga_2_S_3_. Due to the presence of air, this then decomposes further into Ga_2_O_3_. This accounts for the final weight loss of 7% and leaves a final weight of ca. 42%.

UV–Vis diffuse reflectance data (Figure 2d) indicates that the absorption edge for the colourless crystals of the title compound is 4.10(1) eV. This band gap is in the region where the material would be considered to be an insulator and confirms the colourless nature of the crystals. This optical band gap is comparable to those previously reported for open-framework structures of the type [amineH^+^]_6_[Ga_10_S_18_] [13], in which the T3 clusters are linked by their corners.

## 3. Discussion

Traditionally, chalcogenides containing supertetrahedral clusters have been synthesized by high-temperature methods (for purely inorganic solids) or by solvothermal synthesis, in which water or amines are often used as solvents. More recently, the ionothermal method, in which an ionic liquid acts as both the solvent and the structure-directing agent [20], has been successfully exploited for the preparation of chalcogenides [4,21]. Benefits of the ionothermal approach include the ability to carry the synthesis at ambient pressures and the absence of competition between the solvent and the structure directing agent. Ionothermal synthesis has been found to be advantageous for the synthesis of discrete supertetrahedral clusters [4]. It has been suggested that anion-π interactions between chalcogen ions and the organic cations in the ionic liquid may increase the stability of discrete clusters and, under ionothermal conditions, the chalcogens normally at the four corners of the clusters may be easily replaced by the halogens or organic ligands, which reduce the local negative charge and prevent the formation of extended structures [4]. The use of surfactants in the “surfactant-thermal” method [22] offers a potential alternative for the synthesis of supertetrahedra-based clusters, which we have explored in the work presented here. In contrast to the ionothermal method, in the “surfactant-thermal” method reaction mixtures usually contain an amine, which can act as the template, as well as a surfactant, which acts as the solvent. Research to date indicates that surfactants like PEG-400 can also sometimes act as structure-directing agents [22], which are incorporated into the crystal structure of the chalcogenide [23]. While Kanatzidis and co-workers have exploited surfactants in solutions for the preparation of mesostructured phases from T2 [Ge_4_Q_10_]^4−^ clusters (where Q = S, Se) [24], for crystalline chalcogenides the “surfactant-thermal” approach has primarily been applied to thioarsenates [25,26] and thioantimonates [27,28]. Reports of crystalline supertetrahedra-based chalcogenides prepared by the “surfactant-thermal” method appear to be limited to [Mn(en)_2_(H_2_O)][Mn(en)_2_MnGe_3_Se_9_] (where en = ethylenediamine), which contains chains of T2 clusters [29]. To the best of our knowledge, this methodology has not previously been applied to gallium sulfides. The results we present here demonstrate that the “surfactant-thermal” synthetic approach can be exploited for the preparation of discrete supertetrahedral clusters. We speculate that the significantly higher viscosity of PEG-400, when compared to commonly used solvents in solvothermal reactions, might hinder the linkage of discrete supertetrahedral clusters into larger building blocks, and hence favour the crystallisation of discrete units. The cluster described here is the first example of a T3 gallium sulfide supertetrahedral cluster that exists as a discrete unit, with no organic ligands coordinated to the corners, in contrast to many of those produced using ionothermal methods [4]. Previous work on ligand-free supertetrahedral clusters, including [Ga_4_S_8_]^8−^, [In_4_S_8_]^8−^ and [Ge_4_S_10_]^4−^ [8,24], suggests that the cluster reported here is likely to be soluble in highly polar solvents such as water or formamide. Discrete T3 indium sulfide clusters have been previously prepared under solvothermal conditions, by using transition-metal complexes containing π systems, as large countercations [30,31]. By contrast, the approach presented here does not require the use of bulky cations or anion-π interactions to stabilize the clusters. The surfactant, PEG-400, appears to play a key role. Reactions in the absence of PEG-400, where DBU was instead used as the solvent, did not result in the crystallization of the compound reported here.

## 4. Materials and Methods

**Synthesis of [C_9_H_17_N_2_]_6_[Ga_10_S_16_(SH)_4_]:** This compound was prepared using a mixture of Ga metal (133 mg, 2 mmol), thioacetamide (389 mg, 5.2 mmol), 1,8-diazabicyclo[5.4.0]undec-7-ene (DBU, 0.5 mL, 3.35 mmol), and PEG-400 (4 mL). The reaction was performed in a 23 mL Teflon-lined autoclave, which was heated at 140 °C for 6 days. The resulting product was a mixture of colorless crystals of the title compound and unreacted Ga metal.

**Characterization:** As crystals of the title compound degrade during single-crystal data collection, data for this compound were collected at 100 K on a Rigaku FR-E+ Diffractometer, using a rotating anode Mo source. Single-crystal data were collected at the UK National Crystallography Service (Southampton, UK) [32]. The structure was solved using Superflip [33] and refined using the program CRYSTALS [34]. Solvent molecules were located in the Fourier difference maps. The organic DBUH^+^ cations were refined isotropically due to the presence of heavier Ga and S atoms. All hydrogen atoms were added geometrically. Crystallographic data have been deposited with the Cambridge Crystallographic Data Centre as CCDC 2099600, and is also available as Supplementary Materials.

Powder diffraction data were collected using a Bruker D8 Advance powder diffractometer (Cu-Kα1 radiation, λ = 1.54056 Å) for 1 h, over the range 5 ≤ 2θ/° ≤ 60. The sample was fixed to a zero-background holder using a small amount of Vaseline.

Elemental analysis was carried out by MEDAC Ltd. using approximately 3 mg of crystals.

Thermogravimetric analysis was performed using a TA Instruments Q600 TGA on a ground sample, under air.

Diffuse reflectance measurements were carried out using a Perkin Elmer Lambda 35 UV–Vis spectrometer equipped with a diffuse reflectance integrating sphere. BaSO_4_ powder was used as the reference for 100% reflectance.

## 5. Conclusions

We have demonstrated that the use of a surfactant as the solvent facilitates the crystallization of discrete ligand-free T3 clusters of gallium sulfide, which may be attractive as precursors for the synthesis of open-framework sulfides. Further exploratory work on the synthesis of supertetrahedra-based chalcogenides using surfactants as solvents is required to evaluate fully the potential of this synthetic approach.

## Figures and Tables

**Table 1:** Selected crystallographic data for [C_9_H_17_N_2_]_6_[Ga_10_S_16_(SH)_4_].

**Crystallographic Formula**	C_54_H_102_Ga_10_N_12_S_20_
**Mr**	2257.88
**Crystal habit**	Colourless prism
**Crystal system**	Monoclinic
**Space group**	*P*2_1_
***T*/K**	100
***a*/Å**	14.1675(3)
***b*/Å**	14.1898(3)
***c*/Å**	21.3007(4)
***α*/°**	90
***β*/°**	90.5730(18)
***γ*/°**	90
***V*/Å^3^**	4281.95(15)
***Z***	2
***ρ*_cal_/g cm^−1^**	1.751
**Number of parameters**	536
***R*_merge_**	0.0659
***R*(*I* > 3.0*σ*(*I*))**	0.0794
***R*_w_**	0.0595

## Data Availability

Data are contained within the article or Supplementary Materials.

## Supplementary Materials

The following are available online: crystal structure of [C_9_H_17_N_2_]_6_[Ga_10_S_16_(SH)_4_] as a CIF file.
